# Sucrose or glucose compared to breast milk for pain control in preterm infants: a systematic review and meta-analysis

**DOI:** 10.1038/s41372-025-02423-w

**Published:** 2025-10-24

**Authors:** Shaneela Shahid, Jorge Acosta-Reyes, Ivan D. Florez

**Affiliations:** 1 Department of Health Research Methods Evidence and Impact, McMaster University, Hamilton, ON Canada; 2 Department of Pediatrics, McMaster University, Hamilton, ON Canada; 3 Department of Public Health, Universidad del Norte, Barranquilla, Colombia; 4 Department of Pediatrics, University of Antioquia, Medellin, Colombia; 5 Pediatric Intensive Care Unit, Clinica Las Américas-AUNA, Medellin, Colombia; 6 School of Rehabilitation Science, McMaster University, Hamilton, ON Canada

**Keywords:** Outcomes research, Paediatrics, Signs and symptoms

## Abstract

**Abstract:**

The aim of this systematic review/meta-analysis of randomized clinical trials (RCTs) was to determine the efficacy of sucrose or glucose (SG) in preterm infants requiring heel lancing and venipuncture compared to breast milk or expressed breast milk (BM/EBM) for pain control and crying duration. Six RCTs (525 infants) comparing sucrose (24%) or glucose (30%, 10%, 25%) with BM/EBM were included. There was no difference between the alternatives in pain reduction at 30 s post-procedure measured with the Premature Infant Pain Profile (PIPP/PIPP-R) scores (MD -0.95, 95% CI −2.43; 0.54, Low certainty evidence). SG reduced the crying duration (MD –6.88, 95%CI −11.42; −2.34, Moderate certainty evidence). There were no differences between SG and BM/EBM in heart rate change or adverse events. In conclusion, moderate certainty evidence suggests that SG may be superior to BM/EBM for reducing crying duration during heel lancing and venipuncture procedures, but not for reducing pain intensity measured with PIPP/PIPP-R.

**Objective:**

To determine the efficacy of sucrose or glucose (SG) in preterm infants requiring heel lancing and venipuncture compared to breast milk or expressed breast milk (BM/EBM) for pain control and crying duration.

**Study design:**

Systematic review and meta-analysis of randomized clinical trials.

**Results:**

Six RCTs (525 infants) comparing sucrose (24%) or glucose (30%, 10%, 25%) with BM/EBM. There was no difference between the alternatives in pain reduction at 30 s post-procedure measured with the Premature Infant Pain Profile (PIPP/PIPP-R) scores (MD −0.95, 95% CI −2.43; 0.54, Low certainty evidence). SG reduced the crying duration (MD –6.88, 95%CI −11.42; −2.34, Moderate certainty evidence). There were no differences between SG and BM/EBM in heart rate change or adverse events.

**Conclusions:**

Moderate certainty evidence suggests that SG may be superior to BM/EBM for reducing crying duration during heel lancing and venipuncture procedures, but not for reducing pain intensity measured with PIPP/PIPP-R.

## Introduction

Preterm infants are born less than 37 weeks of gestation and are exposed to noise, light, touch, and repeated painful procedures such as venipuncture and heel lancing [1, 2]. Heel lancing and venipuncture are the third and sixth most common painful procedures performed in the neonatal intensive care unit [3, 4]. In infants, these painful procedures occur during an important period of neurodevelopment when the nervous system is vulnerable due to immaturity and neuroplasticity [5]. Preterm infants are more sensitive to pain stimuli as compared to older infants and children due to immature pain inhibition mechanisms at birth, which leads to more distress and delayed recovery from pain in preterm infants [6]. Responses of repeated exposure to painful procedures in preterm infants include increased heart rate [7], oxidative stress [8], and cortisol [9, 10], as well as decreased vagal activity [11], lower weight and head circumference percentiles at 32 weeks of gestation [12, 13], thinner gray matter [14] which, in turn, will be associated with abnormal brain development [13–15]. This may contribute to motor and cognitive developmental delay and behavioral problems of preterm infants in later childhood, such as increased anxiety/stress and attention-deficit disorders, hypervigilance, and exaggerated startle responses [14, 16, 17].

Non-pharmacological interventions are considered feasible alternatives for pain management in term and preterm infants due to the low risk of adverse events [18, 19]. The most studied non-pharmacological interventions to control the pain for these procedures are breast milk, sucrose, or glucose. Although most studies have reported sucrose/glucose (SG) as a non-pharmacological intervention, it could be considered a pharmacological intervention for pain management in neonates (https://www.uptodate.com/contents/prevention-and-treatment-of-neonatalpain? search=pain%20in%20neonates&source=search_result&selectedTitle=1~150&usage_type=default &display_rank=1). It is believed that SG influences the endogenous opioid pathways, which are activated by the sweet taste or analgesic effect through an increase in dopamine and acetylcholine [20, 21]; moreover, the analgesic/calming effects of SG are more gradual and last beyond the end of SG administration [22]. The SG solution is easy to administer and inexpensive, with few side effects, making it possible to use it in infants [23]. SG has been the most common pharmacological intervention that has been well-studied in term infants for pain management [24, 25]. SG has been shown to have a calming effect, decrease the crying duration, and reduce the heart rate compared to a placebo [22, 26–28].

Conversely, breast milk (BM)/expressed breast milk (EBM) is considered a non-pharmacological intervention (https://www.uptodate.com/contents/prevention-and-treatment-of-neonatalpain? search=pain%20in%20neonates&source=search_result&selectedTitle=1~150&usage_type=default &display_rank=1) and is an effective analgesic measure in term and preterm infants [29–34]. Evidence supports that BM/EBM is effective in pain reduction due to painful procedures in neonates when compared with placebo [35–37]. In the last decade, EBM, with or without other non-pharmacological interventions, has been well-studied in preterm infants [34–38].

SG and BM/EBM are effective, safe, and readily available oral interventions that have been well-studied in neonates. Moreover, several systematic reviews have summarized the available evidence of SG and BM/EBM [18, 19, 24, 25, 28, 34–37, 39–43]. As a result, most of the evidence highlights that both interventions are superior to placebo for pain control in neonates. Two recent reviews have highlighted the importance and the need for a synthesis of the evidence on the effectiveness of (SG and BM/EBM) in preterm infants [25, 37], as their comparative efficacy and safety are still being determined in this population. Therefore, we aimed to determine the efficacy and safety of SG compared to BM/EBM in preterm infants requiring heel lancing and venipuncture procedures in terms of pain control and crying duration.

## METHODS

Our systematic review and meta-analysis protocol was registered in PROSPERO (registration number: CRD 42018086917). This report follows the PRISMA - Preferred Reporting Items for Systematic Reviews and Meta-Analyses updated guidelines-2020 [44].

### Eligibility criteria

We included all types of randomized controlled trials (RCTs) examining the effectiveness of sucrose, glucose, or dextrose at any concentration and any dose given orally compared to BM/EBM before venipuncture/heel lancing. The population of interest was preterm infants (25–36 weeks of gestational age) of less than 1 month of postnatal age, who required heel lancing, venipuncture for blood drawing, or venous catheter insertion in any setting.

### Outcomes

The primary outcomes were pain intensity and crying duration. The pain was measured after the venipuncture and heel lancing, and up to 30 s post-procedure, measured by either one of the following pain scales: Premature Infant Pain Profile (PIPP)/PIRR-R (Premature Infant Pain Profile -Revised) [45] and the Comfortneo Pain Scale [46], and Neonatal Pain, Agitation and Sedation Scale (N-PASS) (Neonatal Pain Agitation and Sedation Scale) Scale [47]. We considered both PIPP and PIPP-R scores as scores of both of these scales were highly correlated in both painful and non-painful procedures for infants across all gestational ages (GA) (25–41 weeks) [48, 49]. The total crying duration was defined as the crying duration measured in seconds from the beginning of the venipuncture until its cessation. Secondary outcomes included changes in heart rate (during and after venipuncture), the number of adverse events, and changes in respiratory rate (RR), oxygen saturation, and blood pressure (BP).

### Data sources

We searched MEDLINE and EMBASE via Ovid, the Cochrane Central Register of Controlled Trials (CENTRAL), and CINAHL from inception to April 2024. We did not apply any language restrictions. In MEDLINE, a subject-specific search strategy was combined with the sensitivity-maximizing version of the Cochrane highly-sensitive strategy and modified for other databases (see Appendix). We searched for ongoing trials by searching clinical trials registers, Clinicaltrials.gov, and the World Health Organization (WHO) International Clinical Trials Registry Platform (ICTRP). We followed the standard methods recommended by Cochrane [50].

### Study selection

One author (SS) performed the search. The results from all the databases were merged, and duplicate records were removed using Endnote X8 software [51]. Two reviewers (SS, JAR) independently and in duplicate screened the identified titles/abstracts and full text to assess their eligibility. We included studies for which both reviewers agreed about the eligibility. Disagreements were resolved by a third reviewer (IDF).

### Data abstraction

For each eligible study, two reviewers (SS, JAR) independently and in duplicate, extracted the data into a pilot-tested Microsoft Excel spreadsheet (Microsoft Corporation, 2018). We extracted the following data: participants’ characteristics; risk of bias (RoB) assessment; the number of events and number of patients per arm (dichotomous outcomes) and mean; SD and number of patients per arm (continuous outcomes); for all outcomes of interest.

### Risk of bias

Two reviewers (SS, JAR) independently assessed RoB using the Cochrane RoB tool [50]. The following domains were assessed: sequence generation; allocation concealment; blinding of participants and personnel (performance bias); blinding of outcome assessment (detection bias); completeness of follow-up and selective reporting bias or any other biases, following Cochrane’s recommendations [50].

### Data synthesis and analysis

We conducted pairwise meta-analyses for all the outcomes. Combined effect estimates were reported as risk ratios (RR) for dichotomous outcomes and as mean difference (MD) for continuous outcomes, along with their 95% confidence intervals (CI). For the pain intensity, all included studies used the uniform pain scale PIPP/PIPP-R, except one that used the COMFORTneo as an additional scale and PIPP for pain assessment. We performed meta-analyses using the statistical package Review Manager-V5.3 [52] and applied the generic inverse variance method [53]. We used random-effect models as we assumed heterogeneity among studies, and random-effects models incorporate the heterogeneity in the statistical combination to reflect the uncertainty it may produce around the estimates [50].

We evaluated the statistical heterogeneity with the *Q* statistic and the *I*^2^. We considered substantial heterogeneity present if the *I*^2^ was >50%. A priori, we planned to conduct subgroup analyses based on the mean gestational age (GA) and the SG concentration in the studies. We also planned to conduct a sensitivity analysis to investigate the robustness of our results, excluding the studies with high RoB, for the primary outcomes. We assessed the publication bias visually using a funnel plot [54].

### Assessment of certainty of the evidence

We assessed the certainty of the evidence for each outcome using the Grading of Recommendations Assessment, Development, and Evaluation (GRADE) approach [55], using the online Guidelines Developmental Tool [56] by two reviewers (IDF, JLA) independently and in duplicate. Disagreements were resolved by consensus.

## RESULTS

### Studies selection and description

**Fig. 1:**
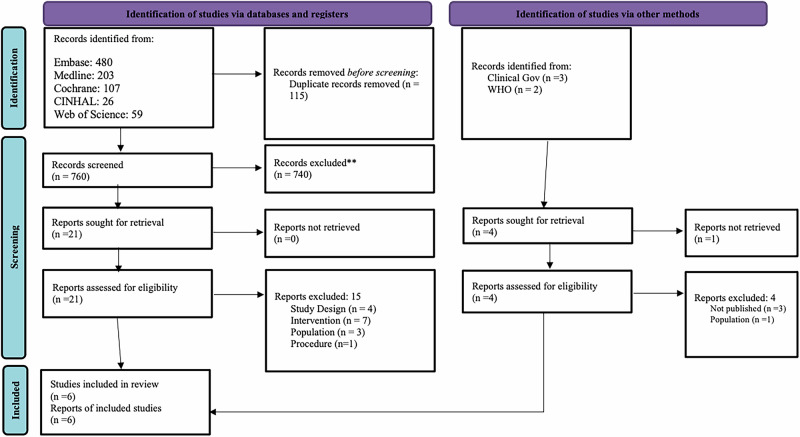
PRISMA Flow chart of study selection. The figure preset the review steps followed to obtain the final included studies.

We identified 875 records from databases and five additional records from other sources. After removing duplicates, 760 records were screened. Twenty-one potentially eligible studies were reviewed in full text. We excluded fifteen studies, provided reasons in the appendix, and included six studies. Figure 1 shows the PRISMA 2020 flow diagram of study selection.

We included 6 RCTs enrolling 525 preterm infants. All studies compared BM/EBM with glucose at different concentrations (30%, 25%, or 10%), or sucrose at 24%. All studies included infants with GA, mostly between 30 and 36 weeks. One study had four arms (glucose 10%, glucose 30%, EBM, and control) [57]. Therefore, for the outcomes this study reported (change in heart rate and crying duration), we combined the information by comparisons: glucose 10% vs. EBM (Skogsdal 1997a) and glucose 30% vs. EBM (Skogsdal 1997b), splitting the sample size of the common comparator in half for each comparison, following the recommendation by Cochrane [50]. Table 1 shows the characteristics of the included studies.

**Table 1:** Summary of the included studies in the systematic review and meta-analysis comparing Sucrose/Glucose vs.

Study	Location	Total number of trial arms and patients randomized	Total Number of Patients	Patient characteristics	Sweet solution – volume, concentration, and time of administration	Comparator – volume, concentration, and time of administration	Outcomes (Effect estimates in mean differences and risk ratio [95%CI])
Bueno 2012	Brazil	2 trial arms (EBM and 25% glucose) 113	88	GA: 34–36 wks. BW: NR PNA: 1–3 days	2 ml of 25% glucose before heel lancing	2 ml of EBM before heel lancing	Pain intensity using PIPP (MD: −3.00 [−4.26, 1.74]) Adverse events (RR 0.48 [0.04, 5.47])
Collados-Gomez 2017	Spain	2 trial arms (EBM/Sucrose) 137	66	GA: <37 wks. BW: <2.5 kg PNA: <14 days	0.1–0.5 ml of 24% sucrose, 2 mins before the procedure	0.1–0.5 ml of EBM, 2 min before the procedure	Pain intensity using PIPP (MD -1.00 [−1.96, −0.04]) Crying duration (MD −6.00 [−11.00, 1.00])
OuYang 2013	Taiwan	3 trial arms	123	GA: <37 wks. BW: NR PNA: <7 days	5 ml of 25% glucose water (glucose) over a 2-min. period, approx. 2 min. before the intervention	5 ml of EBM (obtained from each participant’s mother). over a 2-min. period, approx. 2 min. before the intervention	Included in the meta-analysis: Duration of the first cry: Glucose: 2.0 (IQR 0–45), Milk: 29.5(IQR 0–65) Pain (N-PASS): Glucose: 4.28 (SD 2.61), milk: 4.03 (SD 2.95) HR: Glucose: 151.4 (SD 17), milk: 150.6 (SD 16.3)
Simonse 2012	Netherlands	3 trial arms (BF, EBM, and Sucrose) 71	70	GA: 32–37 wks. BW: 1.6–2800 kg PNA: <14 days	1–2 ml of 24% sucrose before heel lancing	Breast milk with a syringe or breastfeeding before heel lancing, the volume of breast milk was not reported	Pain intensity using PIPP (MD: −0.18 (−2.07, 1.71)
Skogsdal 1997	Sweden	4 trial arms (30% glucose, 10% glucose, EBM, and control)120	90	GA: 25–36 wks. BW: 0.810–3.0 kg PNA: 1–68 days	1 ml of 30% and 10% glucose	1 ml of EBM	Crying duration (MD: −3.70 [−23.99, 16.59]) Heart rate change (MD −6.80 [−14.65, 1.05])
Velumula 2022	USA	2 trial arms (EBM and 24% Sucrose) 94	88	GA: 30–36 wks BW: 1.3–2.5 kg PNA: 1–30 days	2 ml of 24% Sucrose	2 ml of EBM	- Pain (PIPP-R): (MD: 0.30 [−0.11, 0.71]) - Heart Rate: (MD: 1.00 [−2.28, 4.78])

Breast Milk (BM)/Expressed Breast Milk (EBM) in preterm infants.

Studies are sorted in alphabetical order.

*BF* breastfeeding, *BW* birth weight, *EMB* expressed breast milk, *GA* gestational age, *MD* mean difference, *N-PASS* Neonatal Pain, Agitation and Sedation Scale, *PIPP* Premature Infant Pain Profile, *PIPP-R* premature infant pain profile—revised, *PNA* postnatal age, *wks* weeks.

### Risk of bias

**Fig. 2:**
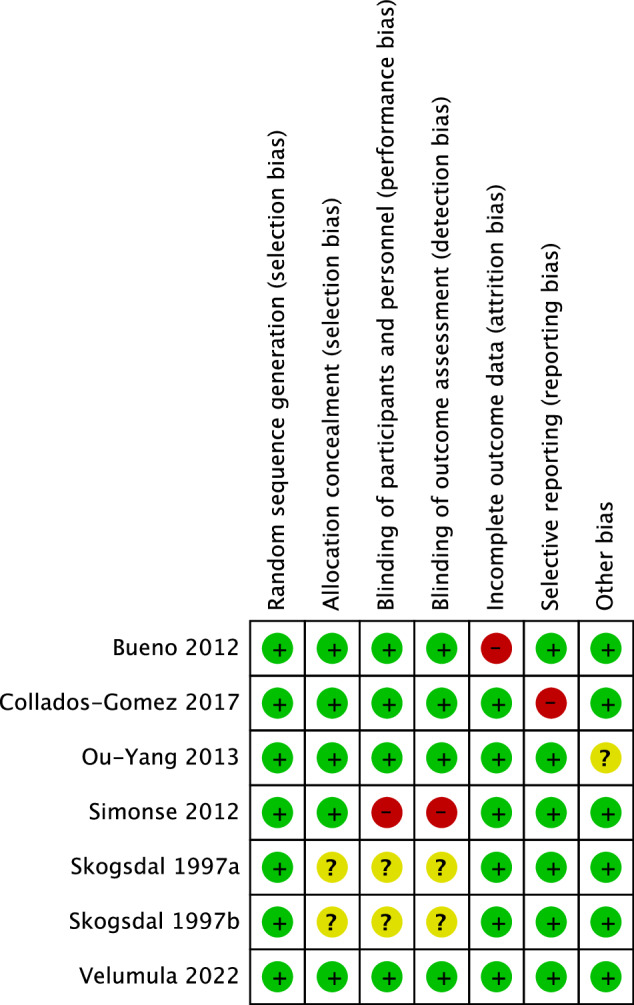
Risk of bias assessment. Summary of risk of bias assessments among the included studies, which includes a review of authors’ judgments on each item for each study.

Figure 2 shows the RoB assessment for each included study. Selection bias was judged to have low RoB in all the studies except Skogsdal [57], which had unclear allocation concealment. One study had issues with performance and detection bias [58]. One study had issues with the follow-up of patients [59], and one study was judged a high risk for reporting bias [60].Velumula had a low RoB in all the domains [61].

### Pain intensity

**Fig. 3:**
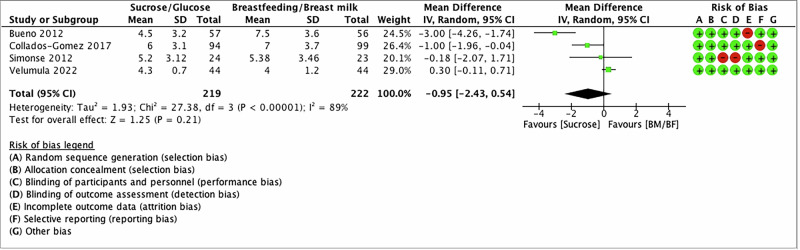
Forest plot for the pain intensity 30 s post-procedure. The forest plot shows the mean difference in the PIPP/PIRR-R scores after the intervention of SG compared to BM/EBM. Horizontal bars denote 95% confidence intervals (95%CIs). Studies are represented as green squares centered on the point estimate of the result of each study. The area of the square represents the weight given to the study in the meta-analysis. The black diamond represents the overall combined estimated effect and its 95%CI. The solid vertical line is the line with no effect.

Five studies reported pain intensity after venipuncture/heel lancing [38, 58–61]. Two studies used only the PIPP scale [59, 60], one study used the N-PASS [38], one study used both the PIPP and ComfortNeo scales [58], and one study used the PIPP-R scale [61]. Therefore, we prioritized statistical combination with PIPP/PIPP-R results; therefore, four studies were included in the analysis. Two studies have provided intensity of pain measured at 30 s after the procedure [60, 61], and one of them [61] reported pain intensity at 30 and 60 s after the procedure. Other studies [58, 59] did not report a specific duration of pain assessment after the procedure. Since there is heterogeneity in reporting the timing of the pain scores assessment among studies, we prioritized the time of pain assessment, which is 30 s post-procedure. The latter was prioritized over 60 s as it might be more clinically relevant as it is the most immediate time for the pain assessment post-procedure. The meta-analysis found that there was no difference in pain scores (when measured 30 s post-procedure) between SG vs BM/EBM groups with substantial heterogeneity (MD −0.95; 95%CI −2.43 to 0.54); *I*^2^ = 89%; Low certainty evidence) (Fig. 3). Table 2 shows the summary of findings—GRADE profile, per outcome.

**Table 2:** Summary of findings table– GRADE Profile.

Sucrose/Glucose compared to Breast Milk/Expressed Breast Milk for Pain Control during Venipuncture or Heel Lance Procedures					
**Patient or population:** Preterm infants undergoing Venipuncture or Heel Lance					
**Setting:** Neonatal Intensive Care Unit					
**Intervention:** Sucrose/Glucose (SG)					
**Comparison:** Breast Milk/Expressed Breast Milk (BM/EBM)					
**Outcomes:** Pain control, crying duration, heart rate, adverse events					
**Outcome № of participant (studies)**	**Relative effect (95% CI)** **(SG in comparison to EBM/BF)**	**Anticipated absolute effects (95% CI)**			**Certainty**
		**SG**	**EBM/BM**	**Absolute Difference** (SG in comparison to EBM/BF)*	
Pain (PIPP/PIPP-R Score) № of participants: 441 (4 RCTs)	-	The mean pain (PIPP Score) was 0	-	MD 0.95 lower (2.43 lower to 0.54 higher)	⨁⨁◯◯ LOW^a,b,c^
Crying Duration (seconds) № of participants: 362 (4 RCTs)	-	The mean Crying duration (seconds) was 0	-	MD 6.88 lower (11.42 lower to 2.34 lower)	⨁⨁⨁◯ MODERATE^d^
Change in Heart Rate № of participants: 167 (2 RCTs)	-	The mean change in Heart Rate was 0	-	MD 1.25 lower (2.28 lower to 4.78 higher)	⨁⨁◯◯ LOW^e,f^
Adverse events № of participants: 113 (1 RCT)	RR 0.79 (0.22 to 2.78)	2.6%	3.3%	7 fewer per 1000 (26 fewer to 59 more)	⨁⨁◯◯ LOW^e,g^

**GRADE Working Group grades of evidence**

**High certainty:** We are very confident that the true effect lies close to that of the estimated effect.

**Moderate certainty:** We are moderately confident in the effect estimate: The true effect is likely to be close to the estimate of the effect, but there is a possibility that it is substantially different.

**Low certainty**: Our confidence in the effect estimate is limited; The true effect may be substantially different from the estimate of the effect.

**Very low certainty:** We have very little confidence in the effect estimate. The true effect is likely to be substantially different from the estimate of the effect.

**Explanations**

^a^Rated down due to risk of bias: one study (Simonse 2012) has high RoB in two criteria (performance and detection biases), and the other two studies had a high risk of attrition bias (Bueno 2012) and of selective reporting (Collados-Gomez 2017).

^b^Rated down due to inconsistency: High heterogeneity (*I*^2^ = 89%).

^c^Rated down to imprecision: a wide 95% CI that includes both a substantial effect (reduction of 2.43 points) and a very low effect (0.54 points).

^d^Rated down due to risk of bias: one study providing 82.5% of weight in the meta-analysis (Collados-Gomez 2017) had a selective reporting outcome, and two studies, providing all together 11.7% of weight had unclear Bias in blinding criteria (Skogdal 1997) and one study (Ou-yang 2013), was considered as unclear RoB in other biases.

^e^Rated down to imprecision: the 95%CI includes both a beneficial effect (reducing heart rate in 11.06 bpm) and harm (increasing heart rate in 1.65 bpm).

^f^Rated down due to risk of bias: only study for this outcome (Skogdal 1997) that had unclear performance and detection bias criteria, and unclear selection bias.

^g^Rated down due to risk of bias: only one study (Bueno 2012) for this outcome, and it has a high risk of attrition bias.

*CI* confidence interval, *MD* mean difference, *RR* risk ratio.

*The risk in the intervention group (and its 95% confidence interval) is based on the assumed risk in the comparison group and the relative effect of the intervention (and its 95% CI).

**Fig. 4:**
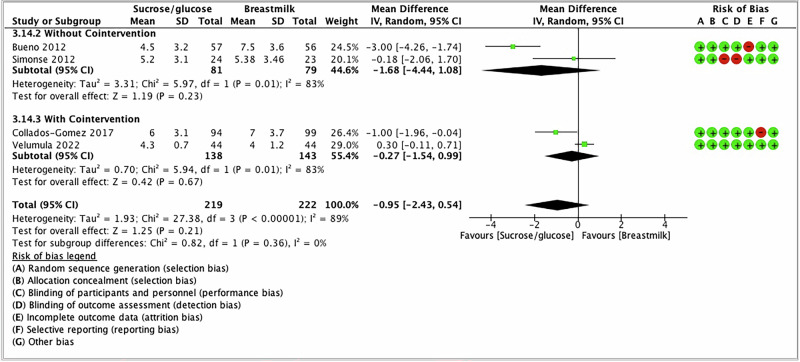
Subgroup analysis—forest plot for the pain intensity based on co-intervention. The forest plot shows the mean difference in the PIPP/PIRR-R scores after the intervention of SG compared to BM/EBM. Horizontal bars denote 95% confidence intervals (95%CIs). Studies are represented as green squares centered on the point estimate of the result of each study. The area of the square represents the weight given to the study in the meta-analysis. The black diamond represents the overall combined estimated effect and its 95%CI. The solid vertical line is the line of no effect.

Two studies have used co-interventions (swaddling ± non-nutritive sucking) along with sucrose and BM/EBM [60, 61], whereas two other included studies did not use any co-interventions [58, 59]. In subgroup analysis based on with or without co-intervention, there was no difference in pain scores between the groups when no co-interventions (MD −1.68; 95% CI −4.44 to 1.08; *I*^2^ = 83%) compared to when the co-interventions were used (MD −0.27; 95% CI −1.54 to 0.99; *I*^2^ = 83%) (Fig. 4).

**Fig. 5:**
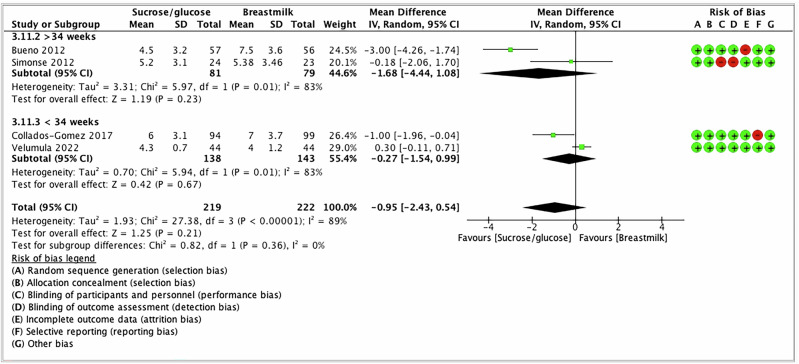
Subgroup analysis—forest plot for the pain intensity based on gestational age. The forest plot shows the mean difference in the PIPP/PIRR-R scores after the intervention of SG compared to BM/EBM. Horizontal bars denote 95% confidence intervals (95%CIs). Studies are represented as green squares centered on the point estimate of the result of each study. The area of the square represents the weight given to the study in the meta-analysis. The black diamond represents the overall combined estimated effect and its 95%CI. The solid vertical line is the line of no effect.

In subgroup analysis based on GA, there was no difference in pain reduction between the groups in studies in infants with more than 34 weeks of gestation (MD −1.68; 95% CI −4.44 to 1.08; *I*^2^ = 83%) when compared with infants less than 34 weeks of gestation (MD −0.27; 95% CI −1.54 to 0.09; *I*^2^ = 83%) (Fig. 5). The only trial [38] using the N-PASS score did not find differences between the interventions.

### Crying duration

**Fig. 6:**
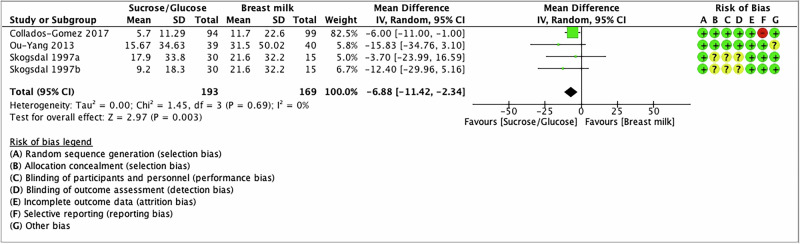
Forest plot for crying duration. The forest plot shows the mean difference in crying duration measured in seconds of SG compared to BM/EBM. Horizontal bars denote 95% confidence intervals (95%CIs). Studies are represented as green squares centered on the point estimate of the result of each study. The area of the square represents the weight given to the study in the meta-analysis. The black diamond represents the overall combined estimated effect and its 95%CI. The solid vertical line is the line of no effect.

Three studies reported total cry duration after venipuncture and heel lancing in 362 preterm infants [38, 57, 60]. Out of three studies, one used 24% sucrose [60], and two used 10%, 25%, and 30% glucose [38, 57]. The meta-analysis showed a reduction in the crying duration with SG compared to BM/EBM (MD −6.88; 95% CI −11.42 to −2.34; *I*^2^ = 0%; moderate certainty evidence) (Fig. 6 and Table 2).

### Heart rate

**Fig. 7:**
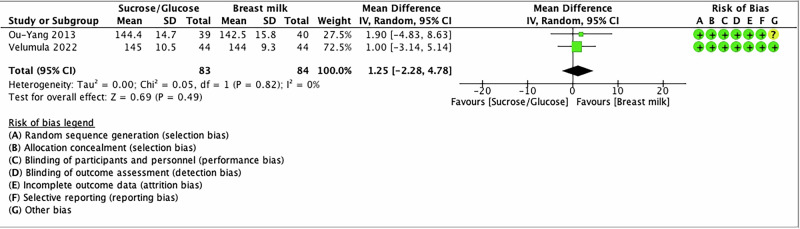
Forest plot for heart rate change. The forest plot shows the mean difference of heart rate change in beats per minute of SG compared to BM/EBM. Horizontal bars denote 95% confidence intervals (95%CIs). Studies are represented as green squares centered on the point estimate of the result of each study. The area of the square represents the weight given to the study in the meta-analysis. The black diamond represents the overall combined estimated effect and its 95%CI. The solid vertical line is the line of no effect.

Two studies compared SG with BM/EBM and reported the outcome of heart rate [38, 61]. The combined estimate found that there was no difference between the groups in the heart rate during heel lancing (MD 1.25; 95% CI −2.28, 4.78, *I*^2^ = 0%; Low certainty evidence) (Fig. 7 and Table 2).

### Adverse events

Four studies reported adverse events [58–61], but only one found events [59]. We found no differences between the interventions (RR 0.79; 95% CI 0.22–2.78; very-low certainty evidence). Adverse events described by Bueno et al. included nausea and/or vomiting and regurgitation, oxygen saturation <80%, and choking [59] (Table 2).

### Other outcomes and analyses

A priori plan was to compare the changes in RR, oxygen saturation, and BP between SG and BM/EBM groups. However, only two of the included studies reported these outcomes [38, 58], Simonse et al. [58] reported only baseline oxygen saturation in all three arms but no information was provided about oxygen saturation during and after the painful procedures. Moreover, this study did not provide any information about RR and BP. Ou-Yang et al. [38] was the only study that provided information about oxygen saturation, RR, and BP baseline, and after the painful procedure, and thus, we did not conduct a meta-analysis for these. We did not generate the funnel plot due to the low number of studies.

### Sensitivity analysis

Due to the heterogeneity in the time of pain assessment post-procedure and the availability of measures at 60 s post-procedure by one of the studies [61], we conducted a sensitivity analysis pooling the results for pain measured at 60 s instead of the 30-s for this study [61]. This analysis found no difference in pain scores between SG vs. BM/EBM groups (MD −0.75, 95%CI −2.60, 1.09), *I*^2^ = 91%; Low certainty evidence) (Appendix—Fig. S1).

The substantial statistical heterogeneity, particularly in pain outcomes, is likely explained by the high RoB (Fig. 2) in three studies included in the meta-analysis (Fig. 3). We found high RoB related to incomplete outcome data [59], selective reporting of the outcome [60], and lack of blinding of participants/personnel/outcome assessment [58]. To further evaluate statistical heterogeneity. We performed a sensitivity analysis for pain outcome was also performed based on the RoB, and three studies with high RoB were excluded [58–60], which left us with one study [61] for pain. We found no difference between the interventions (MD: 0.30; 95%CI −0.11, 0.71) (Appendix—Fig. S2).

Lastly, a sensitivity analysis was performed based on co-intervention for the cry duration. Collados-Gomez used co-intervention [60], as such when this study was removed from the analysis, there was a reduction in crying duration with SG compared to EBM (MD −11.09; 95% CI - −21.91 to −0.16; *I*^2^ = 0%; moderate-certainty evidence) (Appendix—Fig. S3). The same sensitivity analysis for crying duration after excluding one study with high RoB [60] showed that the effect of SG was still significant and larger in comparison to BM/EBM (MD −11.03; 95%CI −21.91 to −0.16, *I*^2^ 0%) (Appendix—Fig. S3).

## Discussion

In this review, we synthesized all the available evidence on SG and BM/EBM efficacy for pain control during venipuncture and heel lancing in preterm infants. We included six studies analyzing 525 preterm infants, and we found that SG might be superior to BM/EBM for the reduction in crying duration in preterm infants, but maybe there is no difference between SG and BM/EBM interventions in pain control in preterm infants when measured with PIPP/PIPP-R.

SG is one of the most frequently studied pharmacological interventions in preterm infants for pain reduction during heel lancing, venipuncture, and intramuscular injections [24]. SG has been recommended by both the American Academy of Pediatrics and the Canadian Pediatric Society to prevent and treat procedural pain in term and preterm infants [62, 63]. Literature on the use of BM/EBM for procedural pain control in preterm infants has evolved in the last decade. SG and BM/EBM have been shown to reduce procedural pain in neonates compared to placebo [18, 24–25, 35–37]. However, as we have described, there are only a handful of trials comparing SG and BM/EBM head-to-head for procedural pain in preterm infants.

We did not find any difference in pain control when SG was compared to BM/EBM in preterm infants; however, SG seems superior to BM/EBM in reducing crying duration in preterm infants. The possible explanation for the superiority of SG in crying duration might be the higher amount of sucrose, which is 240 mg of sucrose per milliliter compared to 66 mg of lactose in one milliliter of premature BM [64]. Moreover, after the sucrose ingestion, the effect of sweetness in the sucrose occurs fast, which is mediated by endogenous opioid release [65]. Additionally, evidence from other studies suggests that lactose does not have a calming effect or reduce the duration of crying when compared to sucrose, fructose, and glucose in human infants [66, 67]. However, this review highlights that SG may not be effective in pain reduction in preterm infants when compared to EBM.

Some challenges and questions need to be answered before we evaluate the effectiveness of sucrose in pain reduction during painful procedures. First, there is variability in the type of sweet solutions. Sucrose seems to be the most common sweet preparation used for pain control in neonates, but dextrose and glucose have also been studied and used [68]. In this review, three out of six trials have used 24% sucrose as an intervention [58, 60, 61], one trial used 30% glucose [57], and two trials used 25% glucose [38, 59]. We planned subgroup analysis based on the concentration, but the low number of studies and variability in reporting the outcome prevented us from conducting it.

One of the key questions that remains to be answered with the use of SG is the best dose and timing of the administration. In literature, most of the studies have reported administering 2 ml of the sweet solution before the painful procedures in term and preterm infants [24, 68], whereas in some studies a lower dose (0.5 ml) of 24% sucrose has been studied for alleviation of pain during venipuncture and heel lancing procedures in preterm infants [69–73]. Moreover, as little as 0.1 ml of 24% sucrose, along with a pacifier, 2 min before a painful procedure, is efficacious in reducing procedural pain in preterm infants [74]. In this review, the volume of sweet solution or BM to be administered was also variable, and the included studies were heterogeneous. Included studies in our review used from 0.1 ml to 5 ml of the sweet solutions. We are unsure how much of this variability may impact the effectiveness. In this review, all the included RCTs had given SG to preterm infants 2 min before the procedure coinciding with the release of endogenous opioids [20, 65].

Crying duration is considered an important patient outcome, and it remains the most widely used indicator for pain intensity in infants [24]. Three of the included RCTs have reported this outcome as total cry duration in seconds [38, 57, 60]. Overall, SG has been shown to reduce the crying duration in preterm infants during painful procedures, which concurs with similar observations in term infants when SG was compared with BM/EBM [75, 76]; however, it was observed that crying duration was longer in preterm infants who had heel lancing [38, 57] as compared to venipuncture [60]. Venipuncture has been reported as less painful than heel lancing in neonates for blood tests [77]. It has been previously reported by Steven et al. that, sick preterm infants have a shorter duration of crying and longer latency to cry during heel lancing procedures when compared to healthy and mildly ill preterm infants [78]; however, we would be able to support this observation where preterm infants who are included in this meta-analysis were healthy preterm infants and were not compared with sick/unstable preterm infants.

Two studies in our review reported a change in heart rate during the heel-lancing procedure and there was no difference in heart rate when SG was compared to BM/EBM in preterm infants during a painful procedure [38, 61]. Further trials on pain treatment in neonates should consider this outcome to be measured more frequently, as HR is a good reflection of the physiological response to pain [79]. Nonetheless, preterm infants do not display physiological indicators as reliably and specifically as term infants [80], thus this outcome needs to be considered along with others such as crying duration.

In the literature, most SG trials have reported adverse events such as gagging, choking, vomiting, and desaturation [24, 25], and very few BM/EBM trials have reported adverse events [35–37]. In this review, four trials reported adverse events but adverse events were noted only in one trial which found no difference between these interventions [59]. It might be fair to say that there are fewer adverse events in both interventions, and these events are considered minor in preterm infants.

Two important systematic reviews on this topic were published in 2023 [25, 37]. Yamada et al. have evaluated sucrose with other non-pharmacological interventions including water /placebo/no intervention, non-nutritive sucking, glucose, breastfeeding, BM, music, acupuncture, facilitated tucking, and skin-to-skin care during heel lancing procedure in both term and preterm infants [25]. The findings of this review concur with our results for the pain outcome which was there was no difference in pain scores when sucrose was compared with EBM in preterm infants. However, the conclusion of Yamada’s review is supported by only one trial in preterm infants comparing SG and BM/EBM [61]. Our review has included more trials in preterm infants as we have directly compared both sucrose and glucose with BM/EBM using PIPP/PIPP-R scales. Moreover, we assessed more than one outcome including duration of crying, heart rate other physiological parameters, and adverse events. We were able to do subgroup analyses based on GA and co-interventions.

Shah et al. conducted a systematic review comparing breastfeeding and EBM with other interventions, including placebo in neonates, specific to term infants who underwent a variety of painful procedures [37]. Moreover, the authors of this review have reported the intensity of pain outcomes using a variety of pain scales, which are commonly used in term infants for various painful procedures. Additionally, Shah’s review only considered studies using the PIPP-R scale to assess the pain outcome in preterm infants, and therefore, the authors were able to include only one trial [61]. Moreover, this review reported the pain outcome in preterm infants at 60 s post-procedure.

Both systematic reviews are of high quality and were very comprehensive; however, they could not provide more specific information on preterm infants and concluded that there was a need for more research on this population. Our review is the first to synthesize the comparative between these two common interventions (SG and BM/EBM) for a particular population (preterm infants) during two common painful procedures using PIPP/PIPP-R scales. Furthermore, our review used pain scores at 30 s post-procedure since it was the most commonly measured time point for pain and considered the most clinically relevant one. Moreover, in our sensitivity analyses, when we pooled the data using the data from the 60-s timepoint of the only study that used it, we also did not find differences between the interventions.

Our systematic review has several strengths. First, we have performed a meta-analysis on an important topic in the preterm population where the two most common interventions were compared, and that has not been studied before. Moreover, this meta-analysis was performed for important patient outcomes such as pain, crying duration, physiological variables, and adverse events. Additionally, we conducted a comprehensive literature search through four databases, gray literature, and manual searches, thereby reducing the risk of publication bias. Since all the included studies have used the PIPP (except one which used PIPP-R) pain scale the results for pain outcomes are presented as a MD, making it easier for clinicians to interpret the results and apply them in their clinical practices. Another important strength of our review is we have included the trials that have used SG and EBM for the two most common needle-related procedures that are performed routinely in these preterm infants. Results of this review are generalizable as heel lancing and venipuncture are common painful procedures in preterm and are routinely performed in both developed and underdeveloped countries. Lastly, we applied high methodological standards in the searches/analyses, following the recommendations by Cochrane [50] and the PRISMA statement 2020 [44] and we used the GRADE approach [55] to assess the certainty of the evidence.

There are a few potential limitations to describe. We could not do some of the important subgroup analyses such as comparing dextrose with BM/EBM due to the low number of RCTs. Our study results apply to specific patient populations(late preterm infants) who are less than 1 month of age and have only a single painful procedure; as such, these results do not apply to sick or extremely preterm infants or preterm infants who have more than one painful procedure. This review could not address the repeated use of SG and BM/EBM in preterm infants and this approach requires further research. These results are key for neonatal units, pediatricians, neonatologists, and nursing staff, as they summarize all the direct evidence from SG vs BM/EBM. Our results will help update hospital protocols for the prevention of needle/related pain in preterm infants.

## CONCLUSION

In conclusion, in preterm infants undergoing venipuncture and heel lancing, compared to BM/EBM, the SG may not reduce pain scores (PIPP/PIPP-R) but very likely reduces the crying duration. We might not be very confident of the pain score results, but we are moderately confident of the results regarding crying duration. Furthermore, we did not find significant adverse effects of the single use of SG, which may be safe in preterm infants. Future high-quality, blinded, randomized, and well-powered trials are needed to address several essential questions in preterm infants related to different sweet solution preparations, repeated doses of SG, and BM for multiple painful procedures. Of particular interest (especially in extremely preterm and sick preterm infants) is the combination of non-pharmacological interventions for pain management in preterm infants; moreover, the long-term effects of these interventions in this patient population should be closely followed and documented.

## Supplementary information

Supplemmentary file

## References

[1] Perroteau A, Nanquette MC, Rousseau A, Renolleau S, Bérard L, Mitanchez D, et al. Efficacy of facilitated tucking combined with non-nutritive sucking on very preterm infants’ pain during the heel-stick procedure: a randomized controlled trial. Int J Nurs Stud. 2018;86:29–35. 29960105 10.1016/j.ijnurstu.2018.06.007

[2] Taddio A, Ohlsson A, Einarson TR, Stevens B, Koren G. A systematic review of lidocaine-prilocaine cream (EMLA) in the treatment of acute pain in neonates. Pediatrics. 1998;101:e1. 9445511 10.1542/peds.101.2.e1

[3] Carbajal R, Rousset A, Danan C, Coquery S, Nolent P, Ducrocq S, et al. Epidemiology and treatment of painful procedures in neonates in intensive care units. JAMA. 2008;300:60–70. 18594041 10.1001/jama.300.1.60

[4] Courtois E, Cimerman P, Dubuche V, Goiset MF, Orfèvre C, Lagarde A, et al. The burden of venipuncture pain in neonatal intensive care units: EPIPPAIN 2, a prospective observational study. Int J Nurs Stud. 2016;57:48–59. 27045564 10.1016/j.ijnurstu.2016.01.014

[5] Williams MD, Lascelles BDX. Early neonatal pain—a review of clinical and experimental implications on painful conditions later in life. Front Pediatr. 2020;8:30. 10.3389/fped.2020.00030 PMC7020755 32117835

[6] Fitzgerald M, Beggs S. Book Review: the neurobiology of pain: Developmental aspects. Neuroscientist. 2001;7:246–57. 11499403 10.1177/107385840100700309

[7] Valeri BO, Gaspardo CM, Martinez FE, Linhares MBM. Pain reactivity in preterm neonates: examining the sex differences. Eur J pain. 2014;18:1431–9. 24733738 10.1002/ejp.508

[8] Slater L, Asmerom Y, Boskovic DS, Bahjri K, Plank MS, Angeles KR, et al. Procedural pain and oxidative stress in premature neonates. J pain. 2012;13:590–7. 22543043 10.1016/j.jpain.2012.03.010 PMC3367033

[9] Grunau RE, Haley DW, Whitfield MF, Weinberg J, Yu W, Thiessen P. Altered basal cortisol levels at 3, 6, 8, and 18 months in infants born at extremely low gestational age. J Pediatr. 2007;150:151–6. 17236892 10.1016/j.jpeds.2006.10.053 PMC1851896

[10] Provenzi L, Giusti L, Fumagalli M, Tasca H, Ciceri F, Menozzi G, et al. Pain-related stress in the Neonatal Intensive Care Unit and salivary cortisol reactivity to socio-emotional stress in 3-month-old very preterm infants. Psychoneuroendocrinology. 2016;72:161–5. 27428089 10.1016/j.psyneuen.2016.07.010

[11] Koenig J, Falvay D, Clamor A, Wagner J, Jarczok MN, Ellis RJ, et al. Pneumogastric (vagus) nerve activity indexed by heart rate variability in chronic pain patients compared to healthy controls: a systematic review and meta-analysis. Pain physician. 2016;19:E55–78. 26752494

[12] Vinall J, Miller SP, Chau V, Brummelte S, Synnes AR, Grunau RE. Neonatal pain in relation to postnatal growth in infants born very preterm. Pain. 2012;153:1374–81. 22704600 10.1016/j.pain.2012.02.007

[13] Smith GC, Gutovich J, Smyser C, Pineda R, Newnham C, Tjoeng TH, et al. Neonatal intensive care unit stress is associated with brain development in preterm infants. Ann Neurol. 2011;70:541–9. 21976396 10.1002/ana.22545 PMC4627473

[14] Brummelte S, Grunau RE, Chau V, Poskitt KJ, Brant R, Vinall J, et al. Procedural pain and brain development in premature newborns. Ann Neurol. 2012;71:385–96. 22374882 10.1002/ana.22267 PMC3760843

[15] Zwicker JG, Grunau RE, Adams E, Chau V, Brant R, Poskitt KJ, et al. Score for Neonatal Acute Physiology–II and neonatal pain predict corticospinal tract development in premature newborns. Pediatr Neurol. 2013;48:123–9. 23337005 10.1016/j.pediatrneurol.2012.10.016 PMC4489879

[16] Grunau RE. Neonatal pain in very preterm infants: long-term effects on brain, neurodevelopment and pain reactivity. Rambam Maimonides Med J. 2013;4:e0025. 10.5041/RMMJ.10132 PMC3820298 24228168

[17] Hall RW, Anand KJS. Short and long-term impact of neonatal pain and stress: more than an ouchie. NeoReviews. 2005;6:e69–75.

[18] Mangat AK, Oei JL, Chen K, Quah-Smith I, Schmölzer GM. A review of non-pharmacological treatments for pain management in newborn infants. Children. 2018;5:130. 30241352 10.3390/children5100130 PMC6210323

[19] Queirós I, Moreira T, Pissarra R, Soares H, Guimarães H. Non-pharmacological management of neonatal pain: a systematic review. Minerva Pediatr. 2022;75:282–95. 10.23736/S2724-5276.22.06871-9 35726765

[20] Blass EM, Ciaramitaro V, Barr RG. A new look at some old mechanisms in human newborns: taste and tactile determinants of state, affect, and action. Monogr Soc Res Child Dev. 1994;59:I–V, 1–81. 8047076

[21] Holsti L, Grunau RE. Considerations for using sucrose to reduce procedural pain in preterm infants. Pediatrics. 2010;125:1042–7. 20403938 10.1542/peds.2009-2445 PMC3047508

[22] Ramenghi LA, Wood CM, Griffith GC, Levene MI. Reduction of pain response in premature infants using intraoral sucrose. Arch Dis Child-Fetal Neonatal Ed. 1996;74:F126–8. 8777660 10.1136/fn.74.2.f126 PMC2528545

[23] Wilson S, Bremner AP, Mathews J, Pearson D. The use of oral sucrose for procedural pain relief in infants up to six months of age: a randomized controlled trial. Pain Manag Nurs. 2013;14:e95–105. 24315282 10.1016/j.pmn.2011.08.002

[24] Cochrane Neonatal Group, Yamada J, Bueno M, Santos L, Haliburton S, Campbell-Yeo M, Stevens B. Sucrose analgesia for heel-lance procedures in neonates. Cochrane Database of Syst Rev. 1996;2023. 10.1002/14651858.CD014806 PMC10466459 37655530

[25] Cochrane Neonatal Group, Yamada J, Bueno M, Santos L, Haliburton S, Campbell-Yeo M, et al. Sucrose analgesia for heel-lance procedures in neonates. Cochrane Database Syst Rev. 2023;8:CD014806. 10.1002/14651858.CD014806 PMC10466459 37655530

[26] Bucher HU, Von Siebenthal K, Keel M, Wolf M, Duc G. Sucrose reduces pain reaction to heel lancing in preterm infants: a placebo-controlled, randomized and masked study. Pediatr Res. 1995;38:332–5. 7494655 10.1203/00006450-199509000-00010

[27] Abad F, Diaz NM, Domenech E, Robayna M, Rico J. Oral sweet solution reduces pain-related behavior in preterm infants. Acta Paediatr. 1996;85:854–8. 8819554 10.1111/j.1651-2227.1996.tb14167.x

[28] Stevens B, Taddio A, Ohlsson A, Einarson T. The efficacy of sucrose for relieving procedural pain in neonates—a systematic review and meta-analysis. Acta Paediatr. 1997;86:837–42. 9307163 10.1111/j.1651-2227.1997.tb08607.x

[29] Cirik VA, Efe E. The effect of expressed breast milk, swaddling and facilitated tucking methods in reducing the pain caused by orogastric tube insertion in preterm infants: a randomized controlled trial. Int J Nurs Stud. 2020;104:103532. 32062050 10.1016/j.ijnurstu.2020.103532

[30] Benoit B, Martin-Misener R, Latimer M, Campbell-Yeo M. Breast-feeding analgesia in infants: an update on the current state of evidence. J Perinat Neonatal Nurs. 2017;31:E2. 10.1097/JPN.0000000000000253 28437305

[31] Baudesson de Chanville A, Brevaut-Malaty V, Garbi A, Tosello B, Baumstarck K, Gire C, et al. Analgesic effect of maternal human milk odor on premature neonates: a randomized controlled trial. J Hum Lact. 2017;33:300–8. 28346843 10.1177/0890334417693225

[32] Holsti L, Oberlander TF, Brant R. Does breastfeeding reduce acute procedural pain in preterm infants in the neonatal intensive care unit? A randomized clinical trial. Pain. 2011;152:2575–81. 22014760 10.1016/j.pain.2011.07.022

[33] Nayak R, Nagaraj KN, Gururaj G (2020). Prevention of Pain during screening for retinopathy of prematurity: a randomized control trial comparing breast milk, 10% dextrose and sterile water. Indian J Pediatr. 2020;87:353–8. 10.1007/s12098-020-03182-6 PMC7223887 31989459

[34] Wu HP, Yin T, Hsieh KH, Lan HY, Feng RC, Chang YC, et al. Integration of different sensory interventions from mother’s breast milk for preterm infant pain during peripheral venipuncture procedures: a prospective randomized controlled trial. J Nurs Scholarsh. 2020;52:75–84. 31762179 10.1111/jnu.12530

[35] Shah PS, Aliwalas L, Shah V. Breastfeeding or breastmilk to alleviate procedural pain in neonates: a systematic review. Breastfeed Med. 2007;2:74–82. 17661578 10.1089/bfm.2006.0031

[36] Shah PS, Herbozo C, Aliwalas LL, Shah VS. Breastfeeding or breast milk for procedural pain in neonates. Cochrane Database Syst Rev. 2012;12:CD00495042. 10.1002/14651858.CD004950.pub3 PMC10108374 23235618

[37] Shah, PS, Torgalkar R, Shah VS. Breastfeeding or breast milk for procedural pain in neonates. Cochrane Database Syst Rev. 2023;8:CD004950. 10.1002/14651858.CD004950.pub4 PMC10464660 37643989

[38] Ou-Yang MC, Chen IL, Chen CC, Chung MY, Chen FS, Huang HC. Expressed breast milk for procedural pain in preterm neonates: a randomized, double-blind, placebo-controlled trial. Acta Paediatr. 2013;102:15–21. 23057434 10.1111/apa.12045

[39] de Vries L. Breastfeeding or breastmilk to alleviate procedural pain in neonates: a systematic review. Breastfeed Rev. 2009;17:29–31. 10.1089/bfm.2006.0031 17661578

[40] Gao H, Gao H, Xu G, Li M, Du S, Li F, et al. Efficacy and safety of repeated oral sucrose for repeated procedural pain in neonates: a systematic review. Int J Nurs Stud. 2016;62:118–25. 27474944 10.1016/j.ijnurstu.2016.07.015

[41] Huang RR, Xie RH, Wen SW, Chen SL, She Q, Liu YN, et al. Sweet solutions for analgesia in neonates in China: a systematic review and meta-analysis. Can J Nurs Res. 2019;51:116–27. 30466313 10.1177/0844562118803756

[42] Stevens B, Ohlsson A. Sucrose for analgesia in newborn infants undergoing painful procedures. Cochrane Database Syst Rev. 2000;CD001069. 10.1002/14651858.cd001069. 10.1002/14651858.CD001069 10796405

[43] Harrison D, Beggs S, Stevens B. Sucrose for procedural pain management in infants. Pediatrics. 2012;130:918–25. 23045554 10.1542/peds.2011-3848

[44] Page MJ, McKenzie JE, Bossuyt PM, Boutron I, Hoffmann TC, Mulrow, CD, et al. (2021). The PRISMA 2020 statement: an updated guideline for reporting systematic reviews. BMJ. 2021;372:n71. 10.1136/bmj.n71 PMC8005924 33782057

[45] Ballantyne M, Stevens B, McAllister M, Dionne K, Jack A. Validation of the premature infant pain profile in the clinical setting. Clin J Pain. 1999;15:297–303. 10617258 10.1097/00002508-199912000-00006

[46] van Dijk M, Roofthooft DW, Anand KJ, Guldemond F, de Graaf J, Simons S, et al. Taking up the challenge of measuring prolonged pain in (premature) neonates: the COMFORTneo scale seems promising. Clin J Pain. 2009;25:607–16. 19692803 10.1097/AJP.0b013e3181a5b52a

[47] Morgan ME, Kukora S, Nemshak M, Shuman CJ. Neonatal Pain, Agitation, and Sedation Scale’s use, reliability, and validity: a systematic review. J Perinatol. 2020;40:1753–63. 33009491 10.1038/s41372-020-00840-7

[48] Stevens BJ, Gibbins S, Yamada J, Dionne K, Lee G, Johnston C, et al. The premature infant pain profile-revised (PIPP-R): initial validation and feasibility. Clin J Pain. 2014;30:238–43. 24503979 10.1097/AJP.0b013e3182906aed

[49] Gibbins S, Stevens BJ, Yamada J, Dionne K, Campbell-Yeo M, Lee G, et al. Validation of the premature infant pain profile-revised (PIPP-R). Early Hum Dev. 2014;90:189–93. 24491511 10.1016/j.earlhumdev.2014.01.005

[50] Higgins JPT, Thomas J, Chandler J, Cumpston M, Li T, Page MJ, Welch VA (editors). Cochrane Handbook for Systematic Reviews of Interventions. 2nd Edition. Chichester (UK): John Wiley & Sons; 2019.

[51] Clarivate Analytics—EndNote X8 2016. http://endnote.com/sites/en/files/m/pdf/en-x8-qrg-mac.pdf.

[52] Cochrane T (2008). Review Manager (RevMan) 5.3. Copenhagen: The Nordic Cochrane Centre.* (No Title),* 373.

[53] Deeks JJ, Higgins JP, Altman DG, Cochrane Statistical Methods Group (2019). Analyzing data and undertaking meta-analyses. Cochrane handbook for systematic reviews of interventions, 241-84.

[54] Lau J, Ioannidis JP, Terrin N, Schmid CH, Olkin I. The case of the misleading funnel plot. Bmj. 2006;333:597–600. 16974018 10.1136/bmj.333.7568.597 PMC1570006

[55] Guyatt G, Oxman AD, Akl EA, Kunz R, Vist G, Brozek J, et al. GRADE guidelines: 1. Introduction—GRADE evidence profiles and summary of findings tables. J Clin Epidemiol. 2011;64:383–94. 21195583 10.1016/j.jclinepi.2010.04.026

[56] GRADEpro G. Computer program on www.gradepro.org Version [July, 2016]. McMaster University. 2014.

[57] Skogsdal Y, Eriksson M, Schollin J. Analgesia in newborns given oral glucose. Acta Paediatr. 1997;86:217–20. 9055897 10.1111/j.1651-2227.1997.tb08872.x

[58] Simonse E, Mulder PG, van Beek RH. Analgesic effect of breast milk versus sucrose for analgesia during heel lance in late preterm infants. Pediatrics. 2012;129:657–63. 22392168 10.1542/peds.2011-2173

[59] Bueno M, Stevens B, Camargo P, Toma E, Krebs V, Kimura A. T281 short-term safety of expressed breast milk and glucose as pain relief strategies for late preterm neonates undergoing heel lancing. Eur J Pain Suppl. 2011;5:59.

[60] Collados-Gómez L, Ferrera-Camacho P, Fernandez-Serrano E, Camacho-Vicente V, Flores-Herrero C, García-Pozo AM, et al. Randomized crossover trial showed that using breast milk or sucrose provided the same analgesic effect in preterm infants of at least 28 weeks. Acta Paediatr. 2018;107:436–41. 29150862 10.1111/apa.14151

[61] Velumula PK, Elbakoush F, Tabb C, Farooqi A, Lulic-Botica M, Jani S, et al. Breast milk vs 24% sucrose for procedural pain relief in preterm neonates: a non-inferiority randomized controlled trial. J Perinatol. 2022;42:914–9. 35197549 10.1038/s41372-022-01352-2

[62] American Academy of Pediatrics, & Fetus and Newborn Committee. Prevention and management of pain in the neonate: an update. Pediatrics. 2006;118:2231–41. 17079598 10.1542/peds.2006-2277

[63] Barrington KJ, Batton DG, MD GF, Wallman C, Canadian Paediatric Society, & Fetus and Newborn Committee. Prevention and management of pain in the neonate: an update. Paediatr. Child Health. 2007;12:137–8. 10.1542/peds.2006-2277 17079598

[64] Lefrak L, Burch K, Caravantes R, Knoerlein K, DeNolf N, Duncan J, et al. Sucrose analgesia: identifying potentially better practices. Pediatrics. 2006;118:S197–202. 17079623 10.1542/peds.2006-0913R

[65] Shide DJ, Blass EM. Opioidlike effects of intraoral infusions of corn oil and polycose on stress reactions in 10-day-old rats. Behav Neurosci. 1989;103:1168. 2558674 10.1037//0735-7044.103.6.1168

[66] Blass EM, Shide DJ. Some comparisons among the calming and pain-relieving effects of sucrose, glucose, fructose and lactose in infant rats. Chem Senses. 1994;19:239–49. 7914461 10.1093/chemse/19.3.239

[67] Blass EM, Smith BA. Differential effects of sucrose, fructose, glucose, and lactose on crying in 1-to 3-day-old human infants: qualitative and quantitative considerations. Dev Psychol. 1992;28:804.

[68] Kassab M, Foster JP, Foureur M, Fowler C (2012). Sweet-tasting solutions for needle-related procedural pain in infants one month to one year of age. Cochrane Database of Syst Rev. 2012;12:CD008411. 10.1002/14651858.CD008411.pub2 PMC6369933 23235662

[69] Elserafy FA, Alsaedi SA, Louwrens J, Sadiq BB, Mersal AY. Oral sucrose and a pacifier for pain relief during simple procedures in preterm infants: a randomized controlled trial. Ann Saudi Med. 2009;29:184–8. 19448377 10.4103/0256-4947.52821 PMC2813645

[70] Biran V, Gourrier E, Cimerman P, Walter-Nicolet E, Mitanchez D, Carbajal R. Analgesic effects of EMLA cream and oral sucrose during venipuncture in preterm infants. Pediatrics. 2011;128:e63–70. 21669894 10.1542/peds.2010-1287

[71] Gaspardo CM, Miyase CI, Chimello JT, Martinez FE, Linhares MBM. Is pain relief equally efficacious and free of side effects with repeated doses of oral sucrose in preterm neonates?. PAIN. 2008;137:16–25. 17854995 10.1016/j.pain.2007.07.032

[72] Asmerom Y, Slater L, Boskovic DS, Bahjri K, Holden MS, Phillips R, et al. Oral sucrose for heel lance increases adenosine triphosphate use and oxidative stress in preterm neonates. J Pediatr. 2013;163:29–35. 23415615 10.1016/j.jpeds.2012.12.088 PMC3687041

[73] Gibbins S, Stevens B, Hodnett E, Pinelli J, Ohlsson A, Darlington G. Efficacy and safety of sucrose for procedural pain relief in preterm and term neonates. Nurs Res. 2002;51:375–82. 12464757 10.1097/00006199-200211000-00005

[74] Stevens B, Yamada J, Beyene J, Gibbins S, Petryshen P, Stinson J, et al. Consistent management of repeated procedural pain with sucrose in preterm neonates: Is it effective and safe for repeated use over time?. Clin J Pain. 2005;21:543–8. 16215340 10.1097/01.ajp.0000149802.46864.e2

[75] Ozdogan T, Akman I, Cebeci D, Bilgen H, Ozek E. Comparison of two doses of breast milk and sucrose during neonatal heel prick. Pediatr Int. 2010;52:175–9. 19627552 10.1111/j.1442-200X.2009.02921.x

[76] Örs R, Özek E, Baysoy G, Cebeci D, Bilgen H, Türküner M, et al. Comparison of sucrose and human milk on pain response in newborns. Eur J Pediatr. 1999;158:63–6. 9950311 10.1007/s004310051011

[77] Larsson BA, Tannfeldt G, Lagercrantz H, Olsson GL. Venipuncture is more effective and less painful than heel lancing for blood tests in neonates. Pediatrics. 1998;101:882–6. 9565419 10.1542/peds.101.5.882

[78] Stevens BJ, Johnston CC, Horton L. Factors that influence the behavioral pain responses of premature infants. Pain. 1994;59:101–9. 7854790 10.1016/0304-3959(94)90053-1

[79] Oliveira NCAC, Gaspardo CM, Linhares MBM. Pain and distress outcomes in infants and children: a systematic review. Braz J Med Biol Res. 2017;50:e5984. 28678920 10.1590/1414-431X20175984 PMC5496157

[80] Maxwell LG, Fraga MV, Malavolta CP. Assessment of pain in the newborn: an update. Clin Perinatol. 2019;46:693–707. 31653303 10.1016/j.clp.2019.08.005

