# Supplementary material for: Sucrose or glucose compared to breast milk for pain control in preterm infants: a systematic review and meta-analysis
Source: J Perinatol. 2025 Oct 24;45(12):1664–74. doi: 10.1038/s41372-025-02423-w (PMC12716988; doi:10.1038/s41372-025-02423-w)

**Appendix**

**Sucrose or Glucose Compared to Breast Milk for Pain Control in Preterm Infants: A Systematic Review and Meta-analysis**

**Database: Embase <1974 to 2024 April 17>**
**Search Strategy:**
**1**  infant*.tw. (551259)
**2**  exp Infant/ (1166595)
**3**  infant.mp. or Infant/ (867104)
**4**  newborn*.tw. (224329)
**5**  newborn.mp. (743259)
**6**  Premature newborn.mp. (1444)
**7**  premature*.tw. (208069)
**8**  Preterm.mp. (138699)
**9**  Neonate.mp. (52910)
**10**  Neonates*.tw. (118458)
**11**  Low Birth weight.mp. or exp low birth weight/ (73312)
**12**  extremely low birth weight infant.mp. or exp extremely low birth weight/ (4570)
**13**  Small for gestational age.mp. or exp small for date infant/ (20053)
**14**  1 or 2 or 3 or 4 or 5 or 6 or 7 or 8 or 9 or 10 or 11 or 12 or 13 (1676534)
**15**  exp Bloodletting/ (417)
**16**  bloodletting.mp. (1044)
**17**  Phlebotom*.tw. (12692)
**18**  phlebotomy.mp. or exp Phlebotomy/ (14900)
**19**  Venipuncture*.tw. (6210)
**20**  Venipuncture.mp. (6079)
**21**  Venepuncture.mp. (1726)
**22**  Blood test.mp. or exp Hematologic Tests/ (362323)
**23**  punctures/ or exp phlebotomy/ (40285)
**24**  Puncture*.tw. (87648)
**25**  Puncture.mp. (115287)
**26**  Vein puncture.mp. or exp vein puncture/ (10158)
**27**  15 or 16 or 17 or 18 or 19 or 20 or 21 or 22 or 23 or 24 or 25 or 26 (498781)
**28**  intravenous catheter.mp. or exp intravenous drug administration/ or exp catheter/ or exp vein catheterization/ or exp peripheral vascular system/ or exp catheterization/ or exp intravenous catheter/ or exp infusion/ (1881996)
**29**  intravenous catheter*.tw. (3737)
**30**  Cannulation.mp. or exp Catheterization/ (245056)
**31**  injection.mp. or exp Injections/ (849095)
**32**  intravenous administration.mp. (45910)
**33**  central venous catheter.mp. or exp central venous catheter/ (38456)
**34**  Percutaneous venous catheter.mp. (9)
**35**  indwelling catheter.mp. or exp indwelling catheter/ (24683)
**36**  injection.mp. or exp injection/ (849095)
**37**  29 or 30 or 31 or 32 or 33 or 34 or 35 or 36 (1172797)
**38**  Heel lancing.mp. (93)
**39**  Heel/ or Heel Prick.mp. (9264)
**40**  Needle related procedure.mp. or Phlebotomy/ (13375)
**41**  Needle related procedure.mp. (16)
**42**  exp Blood Specimen Collection/ (288633)
**43**  Needle Prick.mp. (252)
**44**  exp Infant, Premature/ or Heel lance.mp. (133294)
**45**  Heel lance.mp. (248)
**46**  38 or 39 or 40 or 41 or 42 or 43 or 44 or 45 (441031)
**47**  27 or 37 or 46 (2008650)
**48**  exp SUCROSE/ or Sucrose.mp. (101141)
**49**  Sugar water.mp. (512)
**50**  Dextrose.mp. or exp Glucose/ (519970)
**51**  Glucose water.mp. (262)
**52**  Dextrose water.mp. (165)
**53**  Dextrose 25%.mp. (58)
**54**  glucose.mp. or exp GLUCOSE/ (1052934)
**55**  oral glucose.mp. (53775)
**56**  oral dextrose.mp. (112)
**57**  oral sucrose.mp. (503)
**58**  48 or 49 or 50 or 51 or 52 or 53 or 54 or 55 or 56 or 57 (1141843)
**59**  exp Milk, Human/ (35365)
**60**  Breast milk.mp. (40993)
**61**  Breast feeding.mp. or exp Breast Feeding/ (74781)
**62**  Colostrum.mp. or exp COLOSTRUM/ (11580)
**63**  Expressed breast milk.mp. or exp Breast Milk Expression/ (1127)
**64**  Lactation.mp. or exp LACTATION/ (76372)
**65**  Breastfeeding.mp. (46618)
**66**  Breast-feeding.mp. or Breast Feeding/ (74524)
**67**  exp LACTATION/ or Lactation*.mp. (77574)
**68**  (Breast adj2 feeding).mp. (74933)
**69**  59 or 60 or 61 or 62 or 63 or 64 or 65 or 66 or 67 or 68 (177699)
**70**  Random*.mp. (2336225)
**71**  controlled clinical trial.mp. or exp controlled clinical trial/ (1032660)
**72**  randomized controlled trial/ (817748)
**73**  exp placebo effect/ or exp placebo/ or placebo.mp. (535565)
**74**  crossover$.mp. (122395)
**75**  trial*.mp. or exp "clinical trial (topic)"/ or exp "randomized controlled trial (topic)"/ or exp "controlled clinical trial (topic)"/ (3162027)
**76**  exp factorial design/ or factorial$.mp. (76057)
**77**  group*.mp. (6904664)
**78**  randomly.mp. (575287)
**79**  70 or 71 or 72 or 73 or 74 or 75 or 76 or 77 or 78 (9674137)
**80**  14 and 47 and 58 and 69 and 79 (456)
**81**  limit 80 to yr="2021 -Current" (133)

**Database: Ovid MEDLINE(R) ALL <1946 to April 17, 2024>**
**Search Strategy:**
**1**  infant*.tw. (472223)
**2**  exp Infant/ (1271902)
**3**  infant.mp. or Infant/ (1338183)
**4**  newborn*.tw. (187377)
**5**  newborn.mp. (837381)
**6**  Premature newborn.mp. (1260)
**7**  premature*.tw. (155380)
**8**  Preterm.mp. (97959)
**9**  Neonate.mp. (36779)
**10**  Neonates*.tw. (86526)
**11**  Low Birth weight.mp. or exp low birth weight/ (55999)
**12**  extremely low birth weight infant.mp. or exp extremely low birth weight/ (2442)
**13**  Small for gestational age.mp. or exp small for date infant/ (16663)
**14**  1 or 2 or 3 or 4 or 5 or 6 or 7 or 8 or 9 or 10 or 11 or 12 or 13 (1706109)
**15**  exp Bloodletting/ (2663)
**16**  bloodletting.mp. (3049)
**17**  Phlebotom*.tw. (9287)
**18**  phlebotomy.mp. or exp Phlebotomy/ (8813)
**19**  Venipuncture*.tw. (4182)
**20**  Venipuncture.mp. (4030)
**21**  Venepuncture.mp. (1147)
**22**  Blood test.mp. or exp Hematologic Tests/ (276696)
**23**  punctures/ or exp phlebotomy/ (20926)
**24**  Puncture*.tw. (56624)
**25**  Puncture.mp. (54347)
**26**  Vein puncture.mp. or exp vein puncture/ (625)
**27**  15 or 16 or 17 or 18 or 19 or 20 or 21 or 22 or 23 or 24 or 25 or 26 (358542)
**28**  intravenous catheter.mp. or exp intravenous drug administration/ or exp catheter/ or exp vein catheterization/ or exp peripheral vascular system/ or exp catheterization/ or exp intravenous catheter/ or exp infusion/ (233591)
**29**  intravenous catheter*.tw. (2777)
**30**  Cannulation.mp. or exp Catheterization/ (219928)
**31**  injection.mp. or exp Injections/ (755366)
**32**  intravenous administration.mp. (34920)
**33**  central venous catheter.mp. or exp central venous catheter/ (11748)
**34**  Percutaneous venous catheter.mp. (6)
**35**  indwelling catheter.mp. or exp indwelling catheter/ (21687)
**36**  injection.mp. or exp injection/ (755366)
**37**  29 or 30 or 31 or 32 or 33 or 34 or 35 or 36 (1008860)
**38**  Heel lancing.mp. (74)
**39**  Heel/ or Heel Prick.mp. (4131)
**40**  Needle related procedure.mp. or Phlebotomy/ (3561)
**41**  Needle related procedure.mp. (9)
**42**  exp Blood Specimen Collection/ (16608)
**43**  Needle Prick.mp. (196)
**44**  exp Infant, Premature/ or Heel lance.mp. (66821)
**45**  Heel lance.mp. (212)
**46**  38 or 39 or 40 or 41 or 42 or 43 or 44 or 45 (87222)
**47**  27 or 37 or 46 (1416758)
**48**  exp SUCROSE/ or Sucrose.mp. (91639)
**49**  Sugar water.mp. (379)
**50**  Dextrose.mp. or exp Glucose/ (357158)
**51**  Glucose water.mp. (228)
**52**  Dextrose water.mp. (114)
**53**  Dextrose 25%.mp. (28)
**54**  glucose.mp. or exp GLUCOSE/ (691897)
**55**  oral glucose.mp. (25858)
**56**  oral dextrose.mp. (75)
**57**  oral sucrose.mp. (390)
**58**  48 or 49 or 50 or 51 or 52 or 53 or 54 or 55 or 56 or 57 (778524)
**59**  exp Milk, Human/ (23312)
**60**  Breast milk.mp. (16948)
**61**  Breast feeding.mp. or exp Breast Feeding/ (50899)
**62**  Colostrum.mp. or exp COLOSTRUM/ (10822)
**63**  Expressed breast milk.mp. or exp Breast Milk Expression/ (797)
**64**  Lactation.mp. or exp LACTATION/ (72242)
**65**  Breastfeeding.mp. (37281)
**66**  Breast-feeding.mp. or Breast Feeding/ (50744)
**67**  exp LACTATION/ or Lactation*.mp. (73313)
**68**  (Breast adj2 feeding).mp. (51047)
**69**  59 or 60 or 61 or 62 or 63 or 64 or 65 or 66 or 67 or 68 (150261)
**70**  Random*.mp. (1757564)
**71**  controlled clinical trial.mp. or exp controlled clinical trial/ (709579)
**72**  randomized controlled trial/ (610892)
**73**  exp placebo effect/ or exp placebo/ or placebo.mp. (255719)
**74**  crossover$.mp. (77907)
**75**  trial*.mp. or exp "clinical trial (topic)"/ or exp "randomized controlled trial (topic)"/ or exp "controlled clinical trial (topic)"/ (2118217)
**76**  exp factorial design/ or factorial$.mp. (40032)
**77**  group*.mp. (4903172)
**78**  randomly.mp. (432507)
**79**  70 or 71 or 72 or 73 or 74 or 75 or 76 or 77 or 78 (6791796)
**80**  14 and 47 and 58 and 69 and 79 (145)
**81**  limit 80 to yr="2021 -Current" (19)

**Characteristics of the excluded studies**

| **Study** | **Reason for exclusión** |
| --- | --- |
| Uzelli 2016 | Procedure: RSV immunization |
| Hsieh 2019 | Design: it is not RCT |
| Angeles 2016 | Intervention: No breast milk/feeding, 3 arms: sucrose +NNS, NNS, Placebo |
| Cignacco 2012 | Intervention: No breastfeeding/milk - 3 arms: Sucrose, FT, and Both together |
| Gibbins 2002 | Intervention: No nutritive Sucking, 3 groups: Sucrose, NNS, Sucrose + NNS |
| Bergomi 2014 | Intervention: No breast milk/ breastfeeding, 3 arms: Sucrose, Music, Control |
| Bellieni 2001 | Intervention: 6 groups: Control; Glucose +sucking, sensorial saturation (SS); sucking, oral glucose |
| Baba 2010 | Intervention: No breast milk/Breastfeeding, Group 1: Sucrose +NNS + Vibration, Group 2: sucrose + NNS, No vibration |
| Alemdar 2017 | Intervention: No breastfeeding or Sucrose arms |
| Sahoo 2013 | Population: The mean GA for the preterm infant was 37 weeks |
| Tahir 2021 | Population: The mean GA was 39 weeks |
| Deka 2022 | Population: Both term and preterm infants were included and analysis was done together for both term and preterm infants |
| Queiros 2023 | Design: It is a systematic review |
| Shah 2023 | Design: It is a systematic review |
| Yamada 2023 | Design: It is a systematic review |

FT: Facilitated Tucking, GA; Gestational Age, RSV: Respiratory Syncytial Virus; RCT: Randomized Controlled Trial; NNS: Non-Nutritive Sucking

**Figure S1: Sensitivity Analysis evaluating studies measuring pain at 60 seconds- Forest Plot for Pain intensity 60 seconds post-procedure:** The forest plot shows the mean difference in the PIPP/PIRR-R scores after the intervention of SG compared to BM/EBM. Horizontal bars denote 95% confidence intervals (95%CIs). Studies are represented as green squares centered on the point estimate of the result of each study. The area of the square represents the weight given to the study in the meta-analysis. The black diamond represents the overall combined estimated effect and its 95%CI. The solid vertical line is the line with no effect

**Figure S2. Sensitivity Analysis based on low RoB studies: Forest Plot for Pain Intensity:** The forest plot shows the mean difference in PIRR-R scores after the intervention of SG compared to BM/EBM. Horizontal bars denote 95% confidence intervals (95%CIs). Studies are represented as green squares centered on the point estimate of the result of each study. The area of the square represents the weight given to the study in the meta-analysis. The black diamond represents the overall combined estimated effect and its 95%CI. The solid vertical line is the line of no effect.

**Figure S3. Subgroup/Sensitivity Analysis without co-interventions: Forest Plot for Crying Duration:** The forest plot shows the mean difference in crying duration measured in seconds of SG compared to BM/EBM. Horizontal bars denote 95% confidence intervals (95%CIs). Studies are represented as green squares centered on the point estimate of the result of each study. The area of the square represents the weight given to the study in the meta-analysis. The black diamond represents the overall combined estimated effect and its 95%CI. The solid vertical line is the line of no effect.

**Figure S1: Sensitivity Analysis evaluating studies measuring pain at 60 seconds- Forest Plot for Pain intensity 60 seconds post-procedure**


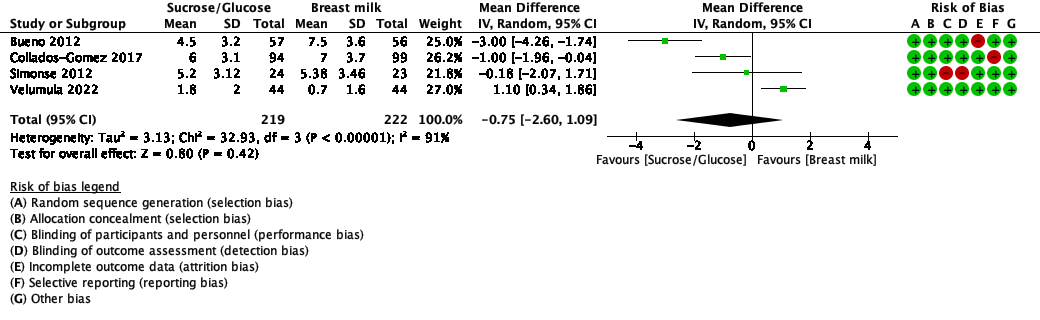


**Figure S2: Sensitivity Analysis based on low RoB studies: Forest Plot for Pain Intensity**


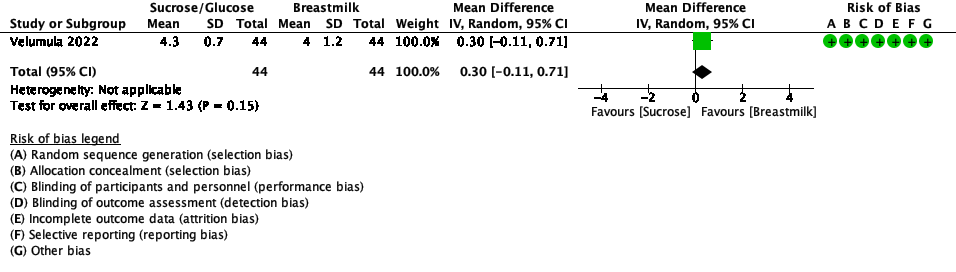


**Figure S3: Subgroup/Sensitivity Analysis without co-interventions: Forest Plot for Crying Duration**


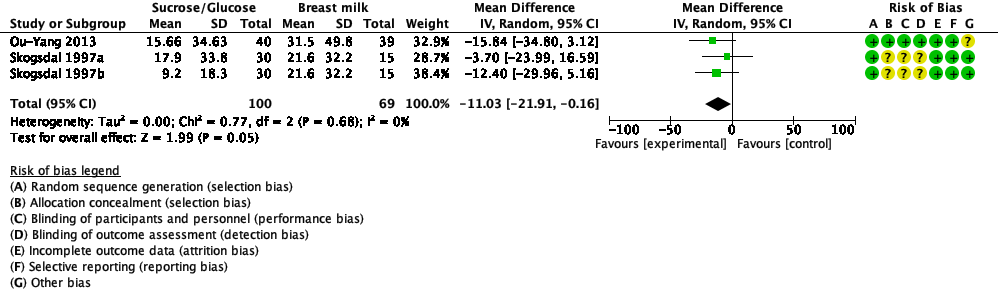

Supplement: Supplementary file 1 — Supplemmentary file [file 41372_2025_2423_MOESM1_ESM.docx]
